# Marketing Animal-Friendly Products: Addressing the Consumer Social Dilemma with Reinforcement Positioning Strategies

**DOI:** 10.3390/ani7120098

**Published:** 2017-12-14

**Authors:** Lenka van Riemsdijk, Paul T.M. Ingenbleek, Hans C.M. van Trijp, Gerrita van der Veen

**Affiliations:** 1 Marketing and Consumer Behaviour Group, Wageningen University, 6708 PB Wageningen, The Netherlands; paul.ingenbleek@wur.nl (P.T.M.I.); hans.vantrijp@wur.nl (H.C.M.v.T.); 2 Research Centre for Innovation in Business and Communication, University of Applied Sciences Utrecht, 3584 BK Utrecht, The Netherlands; gerrita.vanderveen@hu.nl

**Keywords:** animal-friendly products, consumers, marketing, positioning strategies, social dilemma

## Abstract

**Simple Summary:**

Modern production systems aimed at improving animal welfare are more costly than traditional systems. Animal-friendly products are therefore typically more expensive than mainstream products, which presents one of the main barriers to consumer animal-friendly product choice. To overcome this barrier, marketing strategies that associate animal welfare with different types of value, such as taste, healthiness or good feeling, may be useful. This article presents a theoretical framework with marketing strategies using various types of value, suitable for animal-friendly products to encourage consumers to buy animal-friendly instead of mainstream products. We also explain why some consumers, such as those with a rational or an intuitive thinking style, may be more sensitive to some strategies over others, giving directions to marketing managers on how to approach different types of consumers. Because the credibility of animal welfare claims is a critical issue in marketing animal-friendly products, we address this issue as well. Specifically, we propose that, to gain consumer trust, companies selling animal-friendly products need to take into account the impact of their overall strategy on the effectiveness of marketing strategies for individual products and that they may need to collaborate with relevant stakeholders, such as media or animal-interest organizations.

**Abstract:**

This article presents a conceptual framework that aims to encourage consumer animal-friendly product choice by introducing positioning strategies for animal-friendly products. These strategies reinforce the animal welfare with different types of consumption values and can therefore reduce consumers’ social dilemma, which is a major barrier to animal-friendly consumer choices. The article suggests how animal-friendly products can use various types of consumption values (functional, sensory, emotional, social, epistemic and situational) to create an attractive position relative to their competitors. It also explains why some consumer segments, such as those with a specific thinking style, may experience a stronger effect of some strategies, giving directions on how to approach different types of consumers. Finally, building on research asserting that animal welfare is a credence product attribute, the article proposes moderating effects of two factors that help consumers to evaluate the credibility of animal welfare claims, namely corporate social responsibility strategy and the role of stakeholders. Here it concludes that companies selling animal-friendly products need to be aware of the impact of their overall strategy on the effectiveness of positioning strategies for individual products and that, to gain consumer trust, they may need to collaborate with relevant stakeholders, such as media or animal-interest organizations.

## 1. Introduction

In the last two decades, animal welfare has developed into an interdisciplinary field of science, with social scientists playing an important role in advancing our understanding of how the societal concern can translate into improvements in animal welfare in practice (cf. [1]). In that respect, consumer research has a critical position when it comes to improving animal welfare above legal standards in the current market-based policy paradigm. Because consumers ultimately make the decision to accept or reject animal-friendly products, consumer buying behavior presents a powerful drive or a barrier for the development of a market for such products [2]. Existing research on consumer purchase behavior of animal-friendly products has studied, among other topics, consumer concern for animal welfare [3,4], willingness to pay for animal welfare [5,6], the role of consumer trust in animal-friendly labels [7] and the trade-offs that consumers are willing to make between animal welfare and other product benefits, such as healthiness, safety and taste [8,9,10]. The literature has also addressed several institutional and structural barriers, such as the dominant retailing channels and the limited supply of animal-friendly products [11], as well as the transparency of animal-friendly labels [12,13], which, together with consumer purchase behavior, present major challenges to companies selling animal-friendly products. 

While the existing literature provides valuable insights in understanding consumer behavior, these insights are limited in providing guidance in how to design specific marketing instruments for animal-friendly products. In other words, we still know very little on how marketing can encourage consumers to make animal-friendly decisions. As a consequence, there may be unused potential of animal-friendly products in the market. The development of marketing strategies is however complex because marketers must consider the differences in preferences for animal-friendly products that may exist between consumer segments [14]. Such differences may stem from a wide range of factors. Some consumers see purchasing animal-friendly products as an ethical obligation, while others trade it off against price and other product attributes [14,15]. Some perceive animal-friendly products as healthier than mainstream products, while other believe that these products are tastier and of higher quality and even other associate animal welfare with environmental friendliness [16]. Moreover, such differences do not only stem from varying preferences and perceptions but may also stem from norms and values within specific subcultures, such as those linked to the human-animal relations (e.g., whether animals are meant to serve humans) [17], or to the religion (e.g., more concern for certain animal species, such as cows in Hinduism) [4] and even cultural differences at the national level [2,18]. Additionally the structure of and competition in animal-based production sectors and retailing created substantial differences in the habits and preferences of consumers across markets [19]. Next to the challenges pertaining to the differences between consumer segments, marketers should deal in their strategies with competing products and brands that may try to attract consumers with arguments other than animal welfare, like taste or price. Prior research has recognized these elements and referred to them among others as the ethical complex that surrounds animal-friendly products [20] but is not yet grounded these insights in a marketing theoretical framework. We therefore argue that the academic literature on marketing can provide useful insights that may help companies to design strategies for animal-friendly products. Marketing plays however a very small role at best in the current multidisciplinary research field on animal welfare. 

**Figure 1:**
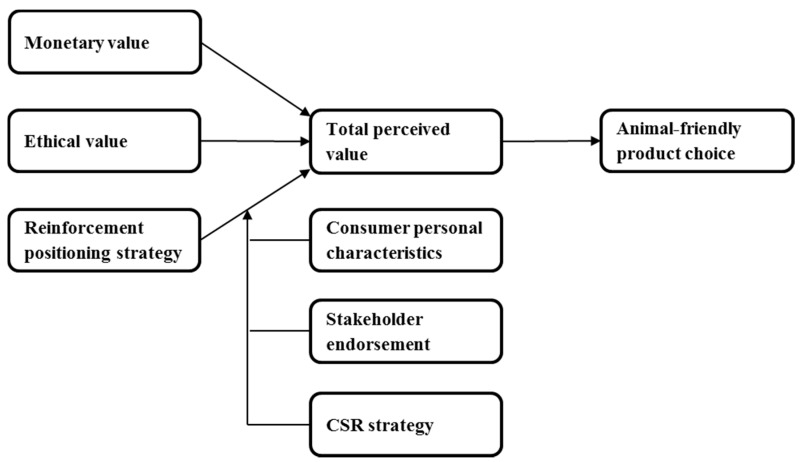
Conceptual framework.

With the present article, we expand the consumer literature on animal-friendly product choice into the marketing domain. We present a conceptual framework (Figure 1) that aims to encourage consumer animal-friendly product choice by introducing marketing strategies for animal-friendly products. In designing specific strategies, we build on social dilemma theory [21], in viewing consumer animal-friendly product choice as a dilemma between maximizing a consumer’s self-interest that includes taste, convenience etc. and the societal interest that includes animal welfare. In recent years, a growing number of scholars has observed that this psychological conflict of interests can be a major barrier for consumers to purchase animal-friendly, or other ethical products [22,23]. They therefore call for marketing strategies that can address such dilemmas. In response to these calls, this article develops a framework in which the social dilemma is addressed by positioning strategies that reinforce the animal welfare with different types of value that consumers may derive from animal-friendly products [24], such as functional (e.g., taste, convenience) or emotional value (e.g., happiness). In short, we theoretically show how animal-friendly products can be made more attractive for consumers who primarily follow their self-interest.

Because consumers differ in their perceptions and preferences for the different positioning strategies, we also propose moderating effects pertaining to consumer personal characteristics. Specifically, we include thinking style, which refers to consumer rationality versus intuitiveness [25] and anthropomorphism, which refers to consumers beliefs in whether animals have feelings, cognition and other humanlike characteristics [26]. Finally, we propose moderating effects of two factors that help consumers to evaluate the credibility of animal welfare claims, namely corporate social responsibility (CSR) strategy [27] and the role of stakeholders [28,29]. In that respect, our framework links to prior studies that look at animal welfare in its socio-economic context [30,31]. In consumer purchase decisions, such factors matter because for consumers, animal welfare is a so called credence attribute: they should trust companies and the system surrounding them that the stated claims are indeed correct [32]. We will formalize the interrelationships between the variables in the conceptual framework with formulas and develop propositions to guide future empirical research. The article will finish with a number of concrete implications for animal-welfare policy makers and managers responsible for the marketing of animal-friendly products.

## 2. The Role of Total Perceived Value in Animal-Friendly Product Choice

To improve animal welfare further through the market, it is vitally important to increase the market shares of animal-friendly products [19]. Hence, animal-friendly product choice is the logical outcome variable in our conceptual framework (see Figure 1). The marketing literature on the determinants of consumer product choice has highlighted several interrelated factors that drive consumer product choice, such as the product’s quality, the product’s total perceived value and customer satisfaction [33,34,35]. A consensus has emerged that the product’s total perceived value [24,33] or, more specifically, the product’s relative perceived value compared to its alternatives [36], is central in predicting consumer product choices. The total perceived value refers to a “consumer’s overall assessment of the utility of a product based on perceptions of what is received and what is given” ([37], p. 14), [38,39]. By comparing several alternatives, the consumer is likely to choose the product that offers the highest total perceived value. In the context of animal-friendly products, the alternatives are typically mainstream products as well as other ethical products, such as fair-trade and organic products. This relationship can be formulated as:

PC_ijk_ = PV_ik_ − PV_jk_ (1)

where:

PC_ijk_ = choice of product *i* (animal-friendly product) over product *j* (alternative product) for consumer *k*.

PV_ik_ (PV_jk_) = total perceived value of product *i (j)* for consumer *k*.

In the consumer behavior literature, total perceived value is typically viewed as the sum of individual consumption values, a concept first introduced by Sheth, Newman et al. [24]. Consumption values include various types (see also Table 1), such as monetary value (the economic sacrifice in the form of prices to be paid), functional value (e.g., product’s healthiness), sensory value (e.g., tastiness), social value (e.g., status) and ethical value, which includes animal welfare [24,40,41]. Because a product typically provides multiple types of value, consumers make complex mental evaluations of the different types of value to assess the total perceived value. For example, in the context of animal-friendly products, consumers typically not only consider the value of animal welfare but they also evaluate the product in terms of its taste, nutritional quality and healthiness (e.g., as in food products), or its design, functional quality and status (e.g., as in fashion clothes). Because each of the consumption values has its unique contribution to the total perceived value, the total perceived value of an animal-friendly product can be broken down into the following formulation:

(2) $${\mathrm{PV}}_{\mathrm{ik}}=\overset{N}{\underset{a=1}{\sum }}{\mathrm{wcv}}_{\mathrm{ka}}*{\mathrm{CV}}_{\mathrm{ika}}$$

where:

a = 1, 2, …., n consumption values.
wcv_ka_ = importance weight given to the *a*-th type of consumption value for consumer *k*, with values ranging from 0 (not important al all) to 1 (very important).
CV_ika_ = perceived level of consumption value *a* of product *i* according to consumer *k*, with values ranging from −1 (much lower than alternative product) to 1 (much higher than alternative product).

As illustrated by Formulas (1) and (2), offering higher total perceived value than the alternative(s) is an important determinant of animal-friendly product choice. Animal friendliness is associated with two types of value. First, animal welfare is an important social issue [42]. Because consumers generally believe that the humane treatment of animals is the right and ethical thing to do [19], animal welfare has a positive impact on a product’s total perceived in terms of its ethical value. Second, the improved animal welfare typically comes with extra costs [43]. These costs are, to a large extent, translated into higher consumer prices [2], having a negative impact on the total perceived value of animal-friendly products in terms of its monetary value. Animal friendly products therefore typically offer higher ethical value but lower monetary value than mainstream products. This presents a critical challenge to the marketing of animal-friendly products.

## 3. Social Dilemma in Animal-Friendly Product Choice

Animal-friendly product choice typically confronts consumers with a social dilemma because they must trade off monetary value against animal welfare (ethical value) when choosing between mainstream and animal-friendly products. (While the paper mainly focuses on consumer decisions between animal-friendly products and mainstream products, the model would also be applicable to decisions between two animal-friendly products of which one has higher animal welfare standards than the other, so the choice would still be influenced by (different levels of) ethical value as well as monetary value.) [22,44]. A *social dilemma* reflects a situation in which the choice of maximizing short-term individual welfare negatively impacts long-term societal welfare [21]. Individual welfare refers to individual benefits, which are enjoyed by an individual consumer or his direct social environment, such as the family, while societal welfare refers to societal benefits, which are shared by a larger social group. The benefits of animal welfare are collectively enjoyed and shared by the society, because better animal welfare arguably affects the mental wellbeing (due to less animal suffering) of both the consumers as well as the non-consumers of animal friendly products [2,42]. Hence, animal welfare can be conceptualized as a societal benefit, also referred to as public benefit in the existing literature (e.g., [21,45]). Monetary value, on the other hand, benefits the buyer himself and can therefore be conceptualized as an individual benefit (or sacrifice). 

In a social dilemma, consumers are faced with a difficult situation because they must compare two essentially different types of benefits—animal welfare and monetary value—and their decision to choose one of those benefits necessarily results in the loss of the other benefit. In such a situation, most consumers are likely to opt for a product that maximizes their individual welfare, rather than a product that maximizes societal welfare. This is because evolution has mostly favored people that have put their immediate self-interest above (long-term) societal interest [23]. In other words, humans have evolved to have a strong tendency to prioritize self-interest over societal interest and to value the present benefits more than those in the future [23]. As long as animal-friendly product choice will present a social dilemma, those tendencies will remain a significant psychological barrier to consumer animal-friendly product choice. This is formalized in the following formula:

(3) $${\mathrm{PC}}_{\mathrm{ijk}}=\overset{N}{\underset{a=1}{\sum }}[\left({\mathrm{wsv}}_{\mathrm{ka}}*\left({\mathrm{SV}}_{\mathrm{ika}}-{\mathrm{SV}}_{\mathrm{jka}}\right)+{\mathrm{wiv}}_{\mathrm{ka}}*\left({\mathrm{IV}}_{\mathrm{ika}}-{\mathrm{IV}}_{\mathrm{ijka}}\right)\right]$$

where:

a = 1, 2, …., n consumption values.

wsv_ka_ = importance weight given to the *a*-th type of societal consumption value (also referred to as societal benefit) for consumer *k*, with values ranging from 0 (not important al all) to 1 (very important).

SV_ika_ (SV_jka_) = perceived level of the *a*-th type of societal consumption value of product *i (j)* according to consumer *k*, with values ranging from −1 (much lower than alternative product) to 1 (much higher than alternative product).

wiv_ka_ = importance weight given to the *a*-th type of individual consumption value (also referred to as individual benefit) for consumer *k*, with values ranging from 0 (not important al all) to 1 (very important).

IV_ika_ (IV_jka_) = perceived level of the *a*-th type of individual consumption value of product *i (j)* according to consumer *k*, with values ranging from −1 (much lower than alternative product) to 1 (much higher than alternative product).

As highlighted in Formula (3), the importance weights consumers give to societal benefits (wsv_ka_) and individual benefits (wiv_ka_) play a crucial role in consumer animal-friendly product choice. As long as animal-friendly products offer lower individual benefits (IV_ika_) than mainstream products (IV_jka_) and higher societal benefits (SV_ika_) than mainstream products (SV_jka_), the choice will depend on the relative differences (e.g., the price premium) and the importance weights. To help consumers to make more animal-friendly product choices, marketing strategies for animal-friendly products can take four forms (In the proposed model, the importance weights given to the societal and individual welfare/benefits are independent from each other, so a consumer could theoretically find societal welfare increasingly important, without any decrease in the importance of his/her individual welfare). First, they can remind consumers of their ethical values at the point of purchase, i.e., increase the importance of societal welfare (wsv_ka_) [46]. This strategy can be executed, for example, by providing a detailed information on the product label on how individual’s product choice impacts the welfare of the animals. Even though this strategy is frequently used in marketing practice, it works against human nature, because it emphasizes societal welfare over individual welfare [23]. Hence, this strategy is mainly useful to target a relatively small segment of consumers who highly value societal welfare and frequently buy animal-friendly products [47]. Second, they can add even more societal benefits to the animal-friendly product to increase the relative difference with its alternative (SV_ika_ − SV_jka_). Companies can do so, for example, by reminding consumers that animal-friendly production has also positive impact on local businesses or natural environment. Similar to the first strategy, this strategy is also mainly useful to target those consumers who put a strong emphasis on societal welfare. Third, decreasing the importance of individual welfare (wiv_ka_) can also be used to increase the total perceived value of animal-friendly products, for example by persuading consumers that, in the case of food that they buy for their families, price should not matter.

Finally, the fourth strategy can reinforce product’s animal friendliness with individual benefits, i.e., decrease the relative difference between individual benefits offered by the animal-friendly product and its alternative (IV_ika_ − IV_jka_) [48]. In this way, the strategy will reduce the social dilemma because consumers will no longer need to trade off societal benefits for individual benefits. This strategy is based on positioning animal welfare as personally relevant and may therefore be more efficient to target the larger consumer segment that prioritizes individual welfare [23]. Such a strategy must reinforce the product’s animal welfare with benefits serving buyer’s individual welfare, as concretized in the reinforcement positioning strategies that we will discuss in the next section. 

## 4. Reinforcement Positioning Strategies for Animal-Friendly Products 

Product positioning is a widely discussed concept in the marketing literature (see, for review, [49]), which is seen as a crucial strategic decision for every company because it determines consumer perception and product choice [50]. *Product positioning* aims to create a clear, unique and desirable position in the minds of target customers [51]. Hence, *positioning strategy* can be defined as a strategic decision to select perceived benefits that create a clear, unique and desirable position relative to the product’s alternatives. *Perceived benefits* refer to actual or potential advantages, such as functional advantages (e.g., nutritional quality, healthiness) or emotional advantages (e.g., happiness, pride) that the customer gains by using the product [52]. The communication of the benefits to the target customers is not a simple one-to-one process. Marketers use product attributes, such as design and animal housing system [52], which are then concretized with package cues and other marketing instruments, such as package color or a certification label. Consumers use these instruments to infer which benefits they can expect from using the product and assess the value and personal relevance of these benefits [53].

The concept of *consumption values* [24] is a valuable instrument that can be used to develop product positioning strategies [54], because it distinguishes between different motives influencing consumer product choice. While the existing literature offers many taxonomies of the concept (for reviews, see [39,40]), it generally agrees that the relevance of different types of consumption values depends on the particular product and particular consumer. As the present paper aims to provide a general guidance for the development of positioning strategies for animal-friendly products, we selected various consumption values, which may be used for different products and consumers. These include six consumption values: functional, sensory, emotional, social, epistemic and situational. In addition, we include *ethical value*, i.e., the product’s capacity to increase societal welfare, in our theoretical model, yet this value is not useful for reinforcement positioning strategies, because it serves societal welfare, rather than individual welfare. Similarly, while *monetary value*, i.e., the economic sacrifice in the form of prices to be paid, is theoretically useful for product positioning strategies, its utility is limited for positioning strategies for animal-friendly products due to the additional costs associated with higher animal welfare.

*Functional value* refers to the “utility derived from the perceived quality and expected performance of the product” ([54], p. 211). The functional reinforcement strategy can, for example, be executed by associating higher animal standards with healthiness, i.e., by positioning free-range eggs as higher in Omega-3 Fatty Acids and lower in saturated fat, thus healthier than alternative products. 

*Sensory value* refers to the product’s appeal to the senses [41]. Positioning on sensory value can, for example, highlight the tastiness of organic beef, which stems from high quality nutrition and slow growth of the animals. 

*Emotional value* stresses the product’s capacity to arouse feelings, moods and emotions [24]. The emotional reinforcement strategy can, for example, be executed by highlighting the natural living environment and happy life or dairy cows living on organic farms, hence eliciting positive emotions in the buyer. 

*Social value* is defined as “the perceived utility acquired from an alternative’s association with one or more specific social groups” ([24], p. 161). Humane treatment of animals is an important issue that our society perceives as the right and ethical thing to do [19], so animal-friendly product choice can be a way to get social acceptance, especially from reference groups that are highly involved in protecting animal welfare. For example, positioning free-range products as the first choice of animal-friendly consumers, emphasizes the social value.

*Epistemic value* refers to a product’s capacity to arouse curiosity or produce intellectual stimulation [55]. Epistemic reinforcement strategy can, for example, make consumers curious by providing interesting package information, together with a QR code with additional details, on a new, innovative husbandry system. 

Finally, *situational value* refers to a specific situation, in which the product becomes more valuable [24]. A positioning strategy reminding consumers of the World Animal Day can use this event to emphasize the importance to buy cosmetics that has not been tested on animals. The examples of package communication for each reinforcement value are listed in Table 1.

By including the proposed consumption values, the total perceived value can be reformulated as follows:

PV_ik_ = β_0_ + wfu_k_ ∗ Fu_ik_ + wse_k_ ∗ Se_ik_ + wem_k_ ∗ Em_ik_ + wso_k_ ∗ So_ik_ + wep_k_ ∗ Ep_ik_ + wmo_k_ ∗ Mo_ik_ + wsi_k_ ∗ Si_ik_ + wet_k_ ∗ Et_ik_ + ε_ik_ (4)

where:

Fu_ik_, Se_ik_, Em_ik_, So_ik_, Ep_ik_, Mo_ik_, Si_ik_, Et_ik_ = perceived functional, sensory, emotional, social, epistemic, monetary, situational and ethical value of product *i* according to consumer *k*, respectively, with values ranging from −1 (much lower than reference product, which is typically the mainstream alternative) to 1 (much higher than reference product).

wfu_k_, wse_k_, wem_k_, wso_k_, wep_k_, wmo_k_, wsi_k_, wet_k_ = importance weight consumer *k* gives to the functional, sensory, emotional, social, epistemic, monetary, situational and ethical value, respectively, with values ranging from 0 (not important al all) to 1 (very important).

**Figure 2:**
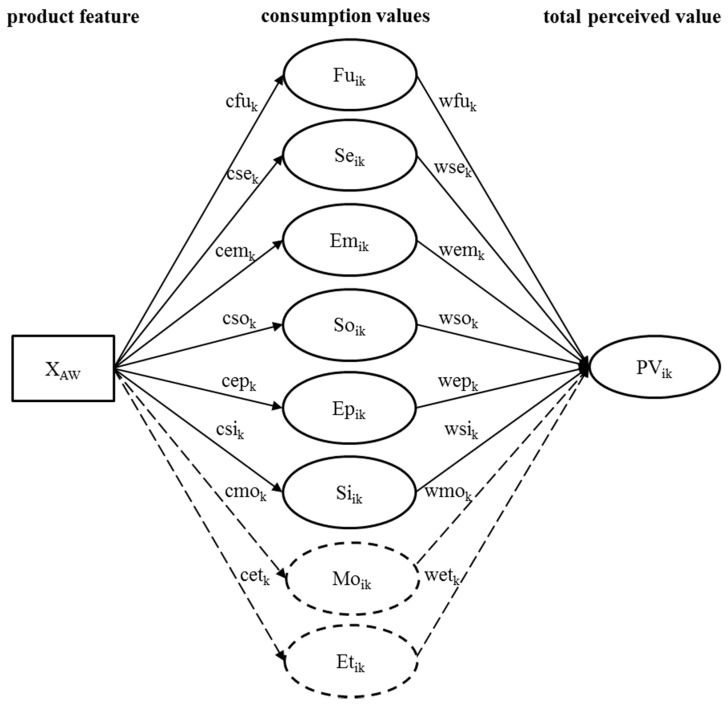
Reinforcement positioning strategies for animal-friendly products (adapted from [56]). The dashed arrows and constructs represent the existing associations of animal welfare with the monetary and the ethical value, which do not represent reinforcement positioning strategy.

As illustrated by Formula (4) and the model depicted in Figure 2, each consumption value impacts the total perceived value. However, to address the social dilemma, marketers need to use those consumption values, individually or combined, that serve the buyer’s individual welfare. Specifically, they need to reinforce product’s animal welfare with consumption values by positioning animal welfare as also being beneficial for one’s individual welfare. Hence we refer to such strategies as *reinforcement positioning strategies*. Such strategies are likely to positively affect consumer animal-friendly product choice, we thus propose:

**Proposition** **1.** *Reinforcement positioning strategy has a positive effect on animal-friendly product choice by increasing the total perceived value of an animal-friendly product*.

The effects of reinforcement positioning strategies can be formulated as:

Effect of functional reinforcement strategy:

Fu_ik_ = β_0_ + cfu_k_ ∗ X_AW_ (5a)

Effect of sensory reinforcement strategy:

Se_ik_ = β_0_ + cse_k_ ∗ X_AW_ (5b)

Effect of emotional reinforcement strategy:

Em_ik_ = β_0_ + cem_k_ ∗ X_AW_ (5c)

Effect of social reinforcement strategy:

So_ik_ = β_0_ + cso_k_ ∗ X_AW_ (5d)

Effect of epistemic reinforcement strategy:

Ep_ik_ = β_0_ + cep_k_ ∗ X_AW_ (5e)

Effect of situational reinforcement strategy:

Si_ik_ = β_0_ + csi_k_ ∗ X_AW_ (5f)

where:

X_AW_ = product feature animal welfare (e.g., a certified label), which can take value 0 (not present) or 1 (present).

cfu_k_, cse_k_, cem_k_, cso_k_, cep_k_, csi_k_, = extent in which consumer *k* associates product’s animal welfare with the functional, sensory, emotional, social, epistemic and situational value, respectively, with values ranging from −1 (strong negative association) to 1 (strong positive association).

Even though reinforcement positioning strategies are likely to have an overall positive effect on consumer animal-friendly product choice, the strength of this effect will be not be the same for all consumers. Some consumer segments, such as those with particular thinking styles [25], may experience a stronger effect of some strategies. In other words, specific personal characteristics are likely to make consumers more sensitive to one strategy over another. In the next section, we will discuss the role of consumer personal characteristics in greater detail.

## 5. Role of Consumer Personal Characteristics in Effectiveness of Reinforcement Positioning Strategies

Different groups of consumers may need different strategies to be persuaded to purchase animal-friendly products. This is because consumer personal characteristics may play an important role in how consumers perceive different reinforcement positioning strategies. Because consumers obviously differ in numerous ways, we restrict ourselves here to two ways that are typical for animal-friendly products, namely thinking style [25] and degree of anthropomorphism [26]. Studies that examined consumers’ attitudes towards animal-friendly products have for example found that consumers can think very differently about such products and the animals at the basis of the production chains (for example about whether animals have feelings and whether they were created to serve humans) [57]. These insights are captured by the two proposed moderator variables discussed below.

### 5.1. Consumer Thinking Style

*Thinking style* is a personality trait that is originally conceptualized as analytic-rational or intuitive-experiential [25,58]. Consumers using an analytic-rational thinking style tend to rely on logical and rational appeals in the decision-making and they like to be intellectually stimulated and challenged [25,59]. Consumers using intuitive-experiential thinking style, on the other hand, typically rely on their intuition and they are more influenced by emotional appeals [59]. 

Thinking style is a two-dimensional construct, consisting of need for cognition and faith in intuition [60]. Existing literature takes different approaches to conceptualizing thinking style based on consumer scores on the two dimensions. Some studies assume the interrelatedness of the dimensions, so they cluster consumers into two groups on the dominant dimension (e.g., [61]), while others assume independence of the dimensions, so they cluster consumers into four groups (e.g., [25]) or study the effects of the dimensions separately [62,63]. The present study proposes to study the individual effects of each dimension, as this may give the most accurate results on how thinking style moderates consumer response to the various reinforcement positioning strategies. 

The proposed reinforcement positioning strategies generally use two types of appeals to reinforce product’s animal welfare: rational and emotional appeals. *Rational appeals* can be defined as those that use logical arguments or reasons related to brand attributes [64]. Rational appeals frequently stress product quality and performance [51]. *Emotional appeals*, on the other hand, aim to make consumer feel good about the purchase, by creating a connection between the consumer and the brand [64]. A free-range meat, for example, can use rational appeal by emphasizing that the product is healthier because it is lower in saturated fat or emotional appeal by making consumer feel good about his/her choice of products that grant good life to the animals. As illustrated in Table 1, some consumption values—functional and epistemic—use rational claims, while others—sensory, emotional, social—use emotional claims (situational value can use both types of claims, as this value is generally tied either to product’s functional or social value [24]. In other words, a product can enhance its situational value in a condition (e.g., an event) in which its purchase or use is more valuable in terms of its functional or social value). Hence, consumers with high need for cognition, who rely on logical and rational appeals in the decision-making, are likely to be more sensitive to strategies using rational appeals to reinforce the ethical value of animal welfare than consumers with low need for cognition. Consumers with high *faith in intuition*, in contrast, are sensitive to emotional messages and hedonic experience, so they will be more sensitive to strategies using emotional appeals to reinforce the ethical value of animal welfare than consumers with low faith in intuition. We thus propose:

**Proposition** **2.** *Need for cognition strengthens the associations between product’s animal welfare and reinforcement values with rational appeals*.

**Proposition** **3.** *Faith in intuition strengthens the associations between product’s animal welfare and reinforcement values with emotional appeals*.

### 5.2. Anthropomorphism

Anthropomorphism, in the context of animal welfare, is the extent to which consumers believe that animals have feelings, cognition and other humanlike characteristics [22,26]. Consumers with a high degree of anthropomorphism arguably believe that animals used for the production of physical products (e.g., food, apparel) or services (e.g., circus performance) should not be seen as products only but rather as live beings who deserve a good life. 

As depicted in Table 1, the proposed reinforcement positioning strategies differ in the types of attributes that they emphasize in that some strategies emphasize *product-related attributes*, i.e., those related to the physical products, while others emphasize *process-related attributes*, i.e., those related to the production process [65]. The first group of strategies includes functional and sensory reinforcement strategy, which emphasize physical product qualities, such as functional quality/healthiness and taste. The second group, including emotional, social and epistemic strategy, emphasizes that due to the husbandry system used in the production process, consumer will get a good feeling, a social approval or an interesting information, so they put the production process central. We expect that consumers with a low degree of anthropomorphism, who thus consider animals as products, will be more sensitive to strategies using product-related attributes. On the other hand, consumers with a high degree of anthropomorphism, who believe that animals are live beings with right to have a good life, will arguably respond stronger to strategies using process-related attributes. We thus propose:

**Proposition** **4.** *Anthropomorphism weakens the associations between product’s animal welfare and reinforcement values with product-related attributes*.

**Proposition** **5.** *Anthropomorphism strengthens the associations between product’s animal welfare and reinforcement values with process-related attributes*.

By including the moderating effects of consumer personal characteristics, the effects of reinforcement positioning strategies can be reformulated as follows:

Effect of functional reinforcement strategy:

Fu_ik_ = β_0_ + cfu_k_ ∗ X_AW_ + ncfu_k_ ∗ X_AW_ ∗ NC_k_ + anfu_k_ ∗ X_AW_ ∗ AN_k_ (6a)

Effect of sensory reinforcement strategy:

Se_ik_ = β_0_ + cse_k_ ∗ X_AW_ + fise_k_ ∗ X_AW_ ∗ FI_k_ + anse_k_ ∗ X_AW_ ∗ AN_k_ (6b)

Effect of emotional reinforcement strategy:

Em_ik_ = β_0_ + cem_k_ ∗ X_AW_ + fiem_k_ ∗ X_AW_ ∗ FI_k_ + anem_k_ ∗ X_AW_ ∗ AN_k_ (6c)

Effect of social reinforcement strategy:

So_ik_ = β_0_ + cso_k_ ∗ X_AW_ + fiso_k_ ∗ X_AW_ ∗ FI_k_ + anso_k_ ∗ X_AW_ ∗ AN_k_ (6d)

Effect of epistemic reinforcement strategy:

Ep_ik_ = β_0_ + cep_k_ ∗ X_AW_ + ncep_k_ ∗ X_AW_ ∗ NC_k_ + anep_k_ ∗ X_AW_ ∗ AN_k_ (6e)

Effect of situational reinforcement strategy:

Si_ik_ = β_0_ + csi_k_ ∗ X_AW_ + ansi_k_ ∗ X_AW_ ∗ AN_k_ (6f)

where:

NC_k_ = level of need for cognition of consumer *k*.

FI_k_ = level of faith in intuition of consumer *k*.

AN_k_ = level of anthropomorphism of consumer *k*.

ncfu_k_, ncep_k_ = effect of need for cognition on the extent in which consumer *k* associates product’s animal welfare (featured with a certified label, for example), with functional and epistemic value, respectively. 

fise_k_, fiem_k_, fiso_k_ = effect of faith in intuition on the extent in which consumer *k* associates product’s animal welfare with sensory, emotional and social value, respectively. 

anfu_k_, anse_k_, anem_k_, anso_k_, anep_k_, ansi_k_ = effect of anthropomorphism on the extent in which consumer *k* associates product’s animal welfare with functional, sensory, emotional, social, epistemic and situational value, respectively.

## 6. Role of Stakeholder Endorsement and CSR Strategy in Effectiveness of Reinforcement Positioning Strategies

Next to consumer personal characteristics, the effectiveness of reinforcement strategies is likely to depend on consumer evaluations of the trustworthiness of the company offering animal-friendly products. The trustworthiness of companies in the animal-based production chains is often debated, not only for reasons of animal welfare but also in the context of animal diseases and impact on human health [66,67]. In some countries, such as in the UK, continuous pressure from non-governmental organizations and media has even led to ‘ethical reform’, through which food retailers were compelled to reconsider their unethical practices [20]. In marketing theoretical terms, animal welfare is a credence attribute, which means that consumers lack the ability to assess whether the product meets the claimed animal welfare criteria or not (like they can with size, color or price) [32]. We therefore include variables in the framework on stakeholder endorsement and the company’s CSR strategy. The first is important because it helps consumers to generate information from an independent source regarding the claimed animal welfare [68]. The second is important because in their CSR strategy companies establish their relationship with society at large [69]. 

### 6.1. Stakeholder Endorsement

For companies selling animal-friendly products, managing communication and support from stakeholders is of crucial and growing importance [29]. Cooperation with stakeholders may therefore be a useful tool that can increase the effectiveness of marketing strategies, primarily when used as a guarantee of the trustworthiness of the product information [44]. While stakeholder definitions and forms of support know many conceptualizations in the current literature (see, for review, [70]), the present study uses *stakeholder endorsement*, defined as a rather passive support from an independent information source [71]. Stakeholder endorsement is a common tool in the marketing strategy that can increase the trustworthiness of the product and its claims [72,73]. Stakeholder endorsement typically involves the use of a certified label, issued by a relevant (international) organization, such as the EU ecolabel issued by the European Union or the cruelty-free bunny label issued by PETA (People for the Ethical Treatment of Animals). 

Stakeholder endorsement may influence the effectiveness of reinforcement positioning strategies because it increases the trustworthiness of the animal welfare claim [29,74]. Reinforcement positioning strategies reinforce product’s animal welfare with consumption values, i.e., position animal welfare as being also beneficial for one’s individual welfare. Hence, the claimed animal welfare is an important element in the reinforcement positioning strategy, because if consumers perceive such claim as untrustworthy, they are unlikely to appraise the claimed individual benefits. These effects have been found in several studies (e.g., [27,28,75]), which commonly conclude that consumer perceptions of the product’s ethical value correlate with the perceptions of other reinforcement values. We thus propose:

**Proposition** **6.** *Stakeholder endorsement strengthens the associations between product’s animal welfare and reinforcement values*.

By including the moderating effects of stakeholder endorsement, the effects of reinforcement positioning strategies can be reformulated as:

Effect of functional reinforcement strategy:

Fu_ik_ = β_0_ + cfu_k_ ∗ X_AW_ + sefu_k_ ∗ X_AW_ ∗ SE (7a)

Effect of sensory reinforcement strategy:

Se_ik_ = β_0_ + cse_k_ ∗ X_AW_ + sese_k_ ∗ X_AW_ ∗ SE (7b)

Effect of emotional reinforcement strategy:

Em_ik_ = β_0_ + cem_k_ ∗ X_AW_ + seem_k_ ∗ X_AW_ ∗ SE (7c)

Effect of social reinforcement strategy:

So_ik_ = β_0_ + cso_k_ ∗ X_AW_ + seso_k_ ∗ X_AW_ ∗ SE (7d)

Effect of epistemic reinforcement strategy:

Ep_ik_ = β_0_ + cep_k_ ∗ X_AW_ + seep_k_ ∗ X_AW_ ∗ SE (7e)

Effect of situational reinforcement strategy:

Si_ik_ = β_0_ + csi_k_ ∗ X_AW_ + sesi_k_ ∗ X_AW_ ∗ SE (7f)

where:

SE = stakeholder endorsement.

sefu_k_, sese_k_, seem_k_, seso_k_, seep_k_, sesi_k_ = effect of stakeholder endorsement on the extent in which consumer *k* associates product’s animal welfare with functional, sensory, emotional, social, epistemic and situational value, respectively.

### 6.2. CSR Strategy

With the growing transparency and access to information that consumers can learn about companies selling animal-friendly products, more emphasis is placed on the company’s image and reputation [76]. The company’s image and reputation therefore play a crucial role in the perceptions and response to the positioning strategies, because consumers tend to compare the information about company’s products with the information about the company. In other words, consumers evaluate and infer the information on specific products from their perceptions of the company [77]. This is particularly important for animal-friendly products, because consumers are not able to validate the product’s animal friendliness themselves. They may therefore use their knowledge of the company’s overall ethical policy to help them decide on whether to trust the animal welfare claims or not. Hence, *Corporate Social Responsibility* (*CSR) strategy*, which refers to company’s activities and approach with regards to its societal obligations [77], is likely to influence the trustworthiness of the animal welfare claim, hence the effectiveness of reinforcement positioning strategies.

While companies can employ various CSR strategies (see, for review, [78]), a common conceptualization distinguishes between proactive and reactive strategies [79,80]. Companies employing reactive CSR strategy “feel they must engage in CSR—mostly unwillingly” ([79], p. 951), while companies employing proactive CSR strategy “actively engage in and support CSR” [80] (p. 641). Building on research that has found proactive CSR strategy being perceived more positive by consumers than reactive strategy [81,82], we expect similar effects with regards to the impact of reinforcement positioning strategies. We thus propose:

**Proposition** **7.** *CSR strategy moderates the strength of the associations between product’s animal welfare and reinforcement values. The associations are stronger for companies employing proactive CSR strategy than for companies employing reactive CSR strategy*.

Finally, by including the moderating effects of CSR strategy, the effects of reinforcement positioning strategies can be reformulated as:

Effect of functional reinforcement strategy:

Fu_ik_ = β_0_ + cfu_k_ ∗ X_AW_ + csfu_k_ ∗ X_AW_ ∗ CS (8a)

Effect of sensory reinforcement strategy:

Se_ik_ = β_0_ + cse_k_ ∗ X_AW_ + csse_k_ ∗ X_AW_ ∗ CS (8b)

Effect of emotional reinforcement strategy:

Em_ik_ = β_0_ + cem_k_ ∗ X_AW_ + csem_k_ ∗ X_AW_ ∗ CS (8c)

Effect of social reinforcement strategy:

So_ik_ = β_0_ + cso_k_ ∗ X_AW_ + csso_k_ ∗ X_AW_ ∗ CS (8d)

Effect of epistemic reinforcement strategy:

Ep_ik_ = β_0_ + cep_k_ ∗ X_AW_ + csep_k_ ∗ X_AW_ ∗ CS (8e)

Effect of situational reinforcement strategy:

Si_ik_ = β_0_ + csi_k_ ∗ X_AW_ + cssi_k_ ∗ X_AW_ ∗ CS (8f)

where:

CS = CSR strategy.

csfu_k_, csse_k_, csem_k_, csso_k_, csep_k_, csec_k_ = effect of CSR strategy on the extent in which consumer *k* associates product’s animal welfare with functional, sensory, emotional, social, epistemic and situational value, respectively.

**Figure 3:**
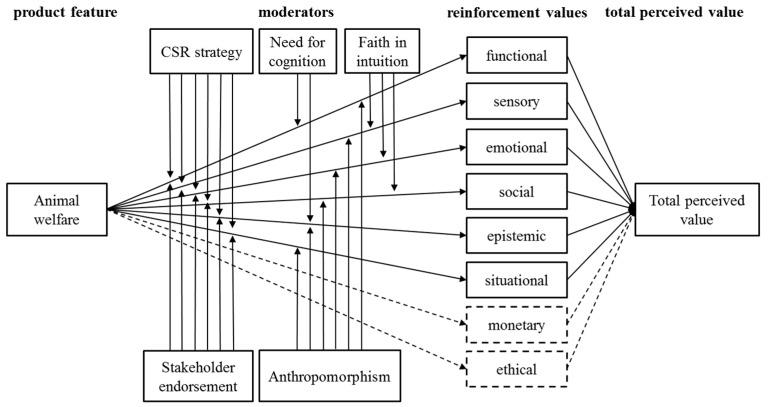
Full conceptual model. The dashed arrows and constructs represent the existing associations of animal welfare with the monetary and the ethical value, which do not represent reinforcement positioning strategy.

The full conceptual model, which includes the moderating effects of consumer need for cognition, faith in intuition and anthropomorphism, as well as the effects of stakeholder endorsement and CSR strategy, is shown in Figure 3.

## 7. Conclusions and Implications

In conclusion, drawing on social dilemma theory, customer value theory and marketing literature on the design of positioning strategies, this article argues that marketing has unused potential to stimulate consumer animal-friendly product choice. When choosing between animal-friendly and mainstream products consumers may be confronted with a social dilemma because they must trade off monetary value against animal welfare. Positioning strategies can reduce the dilemma by reinforcing animal welfare with personally relevant individual benefits. Consequently, consumers who typically opt for mainstream products will be more likely to choose animal-friendly alternatives. 

This conclusion has several implications for marketing managers responsible for the animal-friendly products and animal-welfare policy makers. The main implication from this study logically is that marketing managers should not merely emphasize product’s animal-friendliness through a (certified) label, because this is unlikely to attract consumers who prioritize their self-interest. Instead, they should communicate that animal-friendliness also provides individual benefits, such as taste, healthiness, good feeling, social acceptance etc. While the product package is one way of communicating these benefits, managers can also use other means of communication, such as advertising, in-store displays and company websites.

In designing positioning strategies, managers also need to think deeper about their target customer, i.e., the market segment they would like to attract. This article identified several potential segmentation bases that are relevant for animal-friendly products, namely those based on consumer need for cognition, faith in intuition and anthropomorphism. We argued why these different market segments may be more sensitive to particular positioning strategies, giving practical guidelines to the managers on how to approach these segments. Based on these insights, companies can conduct market research to identify the specific market segments relevant for their products (e.g., age, gender, lifestyle or benefit-related segments). Such research should consider the geographic context to account for country-specific factors, such as cultural and religious influences.

Third, companies may want to think about their collaboration with relevant stakeholders, such as media, animal-interest organizations and consumer organizations. These stakeholders may be helpful, or even necessary, to gain consumer trust in the products, not only with respect to the claimed animal-friendliness but also with respect to other product claims, such as those on product’s healthiness, tastiness, or value for money. The present article mainly focused on stakeholder endorsement, which is a rather passive support typically in form of a certified label, because stakeholder endorsement is particularly suitable for positioning strategies. Companies may, however, also consider other strategies, such as a long-term collaboration with media, or a campaign, in cooperation with an animal-interest organization, communicating the company’s overall contribution to the improvement of animal welfare standards (e.g., the campaign for a global ban on cosmetics animal testing launched by The Body Shop in collaboration with Cruelty Free International).

Fourth, companies need to be aware that their CSR strategy can also influence how consumers perceive positioning strategies designed for individual products. Companies employing a reactive CSR strategy may benefit less from positioning strategies that reinforce animal welfare with individual benefits than companies employing a proactive strategy. Hence, companies need to critically look at their overall CSR strategy, making sure that consumers do not see contradictions between the overall strategy and the positioning strategies for individual products. 

Finally, our theory has implications for policy makers. In the current political-economic environment, policy-makers can stimulate animal-welfare not only by securing the legal lower boundary but also by encouraging private parties in the market to make extra steps by launching animal-friendly products. Obviously, companies will feel more encouraged when the opportunities are clear to them. While in the past many market studies were assigned by policy-makers to investigate citizens’ attitudes towards animal welfare, they may now encourage companies by assigning more studies on which consumers are attracted by which values. Furthermore, policy-makers should secure the interests of companies that genuinely increase standards, by punishing companies that make unjustified animal-welfare claims. In that respect, improving animal welfare through innovation and communication in the market does require government control to ensure that all players follow the rules.

## 8. Limitations and Directions for Future Research

The implementation of the presented framework is limited by several factors. First, as the proposed strategies reinforce improved animal welfare with additional benefits, it is mainly relevant for marketing pre-packed animal-based products with higher levels of animal welfare than the legally set minimum, rather than products without animal ingredients. Second, its applicability is limited to countries where stakeholders, such as non-governmental organizations and media, exist who are powerful and trustworthy enough to influence the perceptions of consumers on animal welfare. Finally, the framework assumes the existence of intensive animal farming industry where consumers are disconnected from animal farming and largely (have to) rely on information that is presented to them through marketing and media.

Our framework suggests several directions for empirical studies examining the propositions formulated in this article. A likely approach to test (parts of) the theory unfolded here is to design consumer experiments that can be conducted in controlled settings. Research may for example test images or actual proto-types of animal-friendly products supported with positioning strategies based on different consumption values. To design the strategies, studies can build on existing research examining the currently-used positioning strategies of animal-friendly products [83], which has shown that if marketers use reinforcement positioning strategies at all, they predominantly rely on strategies emphasizing either emotional value or functional and sensory value. Subsequently the intended or actual product choices can be monitored. Once such studies offer sufficient evidence to bring strategies to the market, researchers can ally with companies by examining the effectiveness of different positioning strategies in a real market context, for example by making use of point-of-purchase data.

Researchers may use similar approaches to test the effects of consumer characteristics. They may measure the different dimensions of thinking style, the level of anthropomorphism and other relevant characteristics, to study their impact on the relationships between positioning strategies and consumers’ product choices. To study these effects, samples with sufficient variance on the relevant consumer characteristics will be needed.

Finally, the effectiveness of one or more strategies can be tested in the context of companies with different CSR policies and/or different levels or support (or even critique) from the side of stakeholders. While such effects can probably be studied most accurate in a controlled experimental setting, stakeholder debates on animal welfare are quite common in many countries in Western Europe and Northern America. We therefore suggest that also case study research on the actual debate and the market responses can make an interesting addition to our understanding of how marketing can help to create a market for products contributing to higher levels of welfare of production animals.

## Figures and Tables

**Table 1:** Examples of package claims for reinforcement positioning strategies for animal-friendly products.

Consumption Value	Objective	Example of Package Claim
functional	associate animal welfare with high functional utility	“Lower in saturated fat and thus healthier due to access to pasture for the animals.”
sensory	associate animal welfare with high sensory experience	“Experience the full taste due to the slow growth and the natural feed.”
emotional	associate animal welfare with positive feelings	“All animals enjoy a happy life with 100% natural environment on our organic farms.”
social	position animal-friendly products as socially accepted or enhancing status	“A growing number of consumers ban battery cages and buy free-range eggs instead.”
epistemic	position animal-friendly products as interesting	“Scan the QR code to see photos and stories from our innovative animal-friendly farms.”
situational	make animal welfare more valuable in a specific situation	“Celebrate the World Animal Day by buying our cruelty-free cosmetics.”
